# Dangers from Chloroform

**Published:** 1898-09

**Authors:** 


### Dangers From Chloroform.

A sad event happened racently in the Catholic hospital at Herne, in Wesphalia. A man who had received a gunshot wound in the abdomen was brought to the hospital, and was, of course, at once operated on. The operation was very difficult and chloroform administration had to be kept up for about four hours. Gas was the illuminant used in the operating room, and it appeared that the gaslight decomposed the chloroform with evolution of powerful chlorinated vapors which overcame the two surgeons and sisters of mercy. One of the sisters died on the second day and the lives of the others were in great danger.—*Lancet.*

"**There** is more in the blood than we have dreamed of in our therapy."— *Whitaker.*

"You can't make a milk punch out of a sow's ear."—*Love.*

---

**Pride Before a Fall.**—Pedagogic father is trying to show off his supposed precocious boy before company.—"My son, who would you rather be, Shakespeare or Dewey?" Little son meditates awhile and says, "I'd rather be Dewey." "What, my son; and why? "Cause he ain't dead."—*New York Weekly.*

---

**As to patenting** the principle of artificial immunizing—the practice has existed amongst the African savages for centuries, of protecting persons against the venom of reptiles by inoculating them with the blood or sweat of the "snake charmer"—a person who has purposely inoculated himself with diluted venom. True, it was empirical; so was the use of quinine in malaria, for many years. Uncle Sam should now give some fellow a patent on vaccine virus.

---

**The Importance of the Role of the Phagocytes** is much enhanced by the new discoveries in regard to their action in tetanus intoxication. They not only destroy the bacteria, but owing to their property of absorbing all the solid particles they encounter, they absorb and render harmless the tetanus toxin if it is combined with a solid substance, such as brain matter. These facts show that the leucocyte is capable of destroying a microbe even when it is secreting a virulent toxin.—*Journal of the American Medical Association.*

---

**The Absorption of Calomel by Leucocytes** after injections into the tissues proceeds very rapidly where the circulating current is rapid and where they reach it before its transformation into mercuric chlorid. In the subcutaneous and muscular tissue the leucocytes merely surround the calomel without absorbing it or carrying it away. The transformation, therefore, only occurs at the point injected, and is promoted by the fluids of the organism. Vaselin holds the calomel in suspension longest and is absorbed by the subcutaneous connective tissue in one minute.—G. Piccardi, *Derm. Cbl.*, April.

